# Regulation of Mammalian Gene Dosage by Long Noncoding RNAs

**DOI:** 10.3390/biom3010124

**Published:** 2013-02-04

**Authors:** Ko-Hsuan Hung, Yang Wang, Jing Crystal Zhao

**Affiliations:** RNA biology program, Sanford-Burnham Medical Research Institute, La Jolla, CA 92037, USA; E-Mails: khung@sanfordburnham.org (K.H.); ywang@sanfordburnham.org (Y.W.)

**Keywords:** epigenetics, lncRNA, genomic imprinting, X-inactivation, *Gtl2*, *Xist*

## Abstract

Recent transcriptome studies suggest that long noncoding RNAs (lncRNAs) are key components of the mammalian genome, and their study has become a new frontier in biomedical research. In fact, lncRNAs in the mammalian genome were identified and studied at particular epigenetic loci, including imprinted loci and X-chromosome inactivation center, at least two decades ago—long before development of high throughput sequencing technology. Since then, researchers have found that lncRNAs play essential roles in various biological processes, mostly during development. Since much of our understanding of lncRNAs originates from our knowledge of these well-established lncRNAs, in this review we will focus on lncRNAs from the X-chromosome inactivation center and the *Dlk1-Dio3* imprinted cluster as examples of lncRNA mechanisms functioning in the epigenetic regulation of mammalian genes.

## 1. Introduction

Long non-coding RNAs are defined as RNAs over 200 nt in length that do not encode proteins. These RNAs were previously considered as transcriptional “noise” or by-products until the development of technologies allowing unbiased high throughput sequencing of transcripts in cells. In 2002, following large-scale sequencing of mouse cDNA libraries, Okazaki *et al*. revealed that a huge proportion of the mammalian transcriptome does not code for proteins and defined lncRNAs as a significant transcript class [1]. The recent ENCODE (Encyclopedia of DNA Elements) study reported over 9640 lncRNA loci in the human genome, roughly half the number of protein-coding genes [2]. These studies have changed our view of the mammalian genome and underscored the importance of understanding lncRNA function at a mechanistic level. Insight into lncRNA function comes mainly from the study of individual lncRNAs, particularly those identified decades ago from specific genomic loci using traditional gene mapping approaches. The most studied lncRNAs are perhaps those transcribed from the X-chromosome inactivation center and from imprinted loci. 

## 2. *Xist* lncRNA and X-Chromosome Inactivation

Female mammals have two X-chromosomes and therefore potentially a two-fold excess of X-linked genes relative to XY males. Such an imbalance in X-linked genes would lead to female early embryonic lethality. To overcome this problem, female mammals silence one (Xi) of the two X chromosomes to equalize X-linked gene dosages between the sexes during early development, a process known as X-chromosome inactivation (XCI) [3,4]. A large body of literature exists relevant to different types of XCI (imprinted *vs*. random), how XCI evolved, and the involvement of XCI in cancer development. Those topics are reviewed elsewhere [5,6,7,8,9]; here, we focus on the role played by lncRNAs in XCI. 

### 2.1. The X Inactivation Center and Xist lncRNA

**Figure 1:**
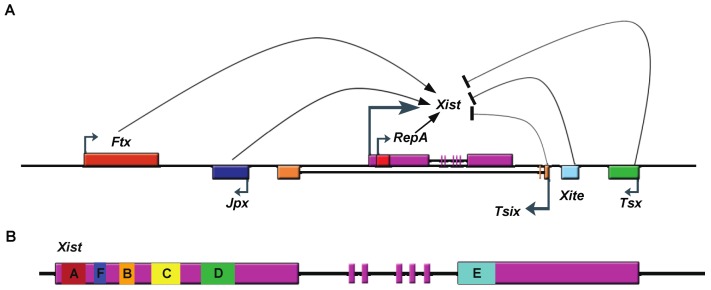
Schematic drawing of the Xic and of *Xist*. A. lncRNAs transcribed from Xic, a region of the X-chromosome necessary and sufficient to trigger XCI. These lncRNAs include *Xist* (X-inactivation specific transcript), *Tsix* (antisense of Xist), *Tsx* (testis-specific X-linked gene), *Xite* (X-inactivation intergenic transcription element), *RepA* RNA, *Jpx* (also known as *Enox* (Expressed Neighbor of *Xist*)), and *Ftx* (Five prime to *Xist*). *Ftx*, *JPX*, and *RepA* lncRNAs promote *Xist* transcription, while *Tsix*, *Xite* and *Tsx* inhibit it. B. Gene structure of *Xist* RNA showing location of tandem repeats (A-F). Exons are represented as boxes.

Early investigators discovered an X-linked locus, the X chromosome inactivation center (Xic), required to trigger X chromosome inactivation [10,11,12]. Amazingly, this center is enriched with lncRNAs, most of them functioning in XCI (Figure 1A). The best-studied and most important of these is the 17Kb X-inactivation specific transcript, known as *Xist*, which is expressed exclusively from the inactivated X-chromosome and essential for the establishment of XCI in early development [13,14,15]. XCI is arbitrarily divided into three stages. At the pre-XCI stage, *Xist* is expressed at low levels from two active X-chromosomes. During the initiation stage of XCI, *Xist* is upregulated and begins to “spread” along one of the X-chromosomes and converts that allele into heterochromatin characterized by: (1) genome-wide loss of euchromatic marks; (2) gain of heterochromatic marks; (3) increased DNA methylation; and (4) silencing of gene expression. This very dynamic stage requires *Xist*. Upon establishment of XCI, the inactive X remains silenced throughout subsequent cell divisions. During this maintenance stage, *Xist* remains highly expressed from the inactive X. However, deletion of *Xist* RNA does not result in chromosome-wide gene reactivation [16,17,18]. Therefore, *Xist* RNA is not required at this stage of XCI [19]. 

### 2.2. Xist RNA Contains Multiple Functional Domains

Like proteins, lncRNA also exhibits functional domains. *Xist* RNA contains six different repeat regions (A to F) (Figure 1B). The most well studied is the repeat A region located at the *Xist* RNA 5’-end [20]. Transgene studies suggest that *Xist* lacking the repeat A region cannot initiate gene silencing [20]. The function of that region was subsequently investigated *in vivo* using a gene targeting approach. Surprisingly, deletion of the A-repeats altered regulation of the mutant *Xist* allele such that it was not expressed [21], making analysis of that deficiency difficult *in vivo*. *Ex vivo* studies using mouse embryonic stem cells (mESCs) revealed at least two independent mechanisms for the silencing function of the A-repeats. One study suggests that the region is required for proper splicing of *Xist* RNA by directly interacting with splicing factor ASF/SF2 [22]. In another study, we discovered that a shorter transcript within the *Xist* locus is transcribed through the RepA region in mESCs and we named it *RepA* RNA [23]. Knockdown of that *RepA* transcript drastically decreased *Xist* levels, similar to the phenotype observed following *in vivo* deletion of the repeat A region of *Xist*, suggesting that *RepA* RNA is an important regulator of *Xist* expression. We found that both *Xist* and *RepA* RNA can bind the chromatin repressor Polycomb repressive complex 2 (PRC2) through repeat A region and such interactions are required for the establishment of the chromosome-wide H3K27-3me heterochromatic mark during the initiation of XCI. 

Several laboratories have analyzed the structure of the repeat A domain in order to understand the molecular basis for RNA-protein interaction. The A-repeat region contains 7.5–8.5 tandem repeats (variable among different species) of a conserved ~26-mer sequence. Computational analysis indicates that each repeat has a double stem loop structure [20]. However, NMR studies of an *in vitro* transcribed 26-mer showed that only the first predicted hairpin is formed, while the second predicted hairpin mediates duplex formation among different repeats [24,25]. One limitation of NMR studies is that only a short sequence, such as one of the A repeats, can be analyzed. By carrying out a FRET experiment using chemical and enzymatic probes, another study examined the structure of the whole domain and found that the A region contains two long stem-loop structures, each including four repeats [26]. These studies highlighted the importance of RNA’s structure for its function. 

Unlike A repeats, the structure and function of the other *Xist* repeats are not well understood. Nevertheless, Repeat regions C and F have shown to regulate *Xist* RNA localization, potentially through interacting with the transcription factor YY1 [20,27,28,29].

### 2.3. Upregulation of Xist RNA during XCI Initiation

Since *Xist* upregulation is crucial for XCI, much effort has been made to elucidate cis-elements and trans-acting factors that control its expression. The following sections describe how lncRNAs and pluripotency factors regulate *Xist* expression.

#### 2.3.1. LncRNAs

In addition to *Xist*, the XIC harbors several lncRNAs (Figure 1A). One of them, *Tsix*, is transcribed antisense to *Xist* and covers the entire *Xist* locus [30,31,32,33,34]. At pre-XCI stage, *Tsix* expression exceeds that of *Xist*. When *Tsix* is deleted, *Xist* is non-randomly upregulated from the disrupted allele, suggesting that *Tsix* represses *Xist* expression. Several mechanisms have been proposed to explain this activity. One is that *Tsix* modulates the chromatin state of the *Xist* promoter. Evidence supporting this hypothesis is that the *Xist* promoter exists in different chromatin states in wildtype cells versus cells expressing *Tsix* truncation constructs [35,36,37], and that *Tsix* lacking the last exon, which overlaps with the *Xist* promoter region, fails to regulate *Xist* [38]. Another possible mechanism is that *Tsix* forms an RNA duplex with *Xist* and regulates it through the RNAi pathway [39]. Although Dicer and *Tsix* reportedly regulate *Xist* synergistically in mESCs [39], no microRNA has yet been identified from the *Xist*/*Tsix* locus. We found that *Tsix* RNA can also bind the PRC2 complex and have proposed that *Tsix* controls *Xist* activity by titrating away *Xist*-interacting proteins [23]. Furthermore, *Tsix* couples with several proteins acting as pluripotency factors in early embryos and ESCs to maintain low expression of *Xist* [40]. (This mechanism is discussed below.) In addition to *Tsix*, other studies have shown that lncRNAs *Xite* (X-Inactivation Intergenic Transcription Element) [41] and *Tsx* (Testis Specific X-linked Gene) [42] repress *Xist* expression through *Tsix* activation, while *Jpx* (also known as *Enox* (Expressed Neighbor of Xist)) [43] and *Ftx* (Five prime to Xist) [44] activates *Xist* RNA through unknown mechanisms. 

#### 2.3.2. Pluripotency Factors

Since undifferentiated female ESCs have two active X chromosomes and express low levels of *Xist* from both, it has long been thought that X-inactivation is coupled to the pluripotency state of mESCs. Recent studies support this hypothesis by showing that pluripotent factors play a repressive role in *Xist* regulation. Conditional knockout of Oct4 (also known as Pou5f1) or Nanog in male mESCs, where *Xist* is expressed at low levels and then silenced after differentiation, results in unexpected *Xist* up-regulation during differentiation [45]. Furthermore, coating of both X chromosomes with *Xist* is seen in female differentiating ES cells following Oct4 knockdown [46]. The first intron of the *Xist* locus exhibits binding sites for Oct4, Nanog, and Sox2 [45,47,48]. However, deletion of intron1 results in a small increase in *Xist* expression, suggesting these factors may alter *Xist* function by binding other genetic elements [49]. Indeed, Oct4 also directly binds and activates *Tsix* and *Xite* to repress *Xist* [46]. In addition to the pluripotency factors noted above, a recent study found the protein encoded by the newly identified pluripotency gene Prdm14 also represses *Xist* RNA expression [47]. 

Interestingly, while one set of pluripotency factors represses *Xist*, a different set of factors activates *Tsix*. For example, expression of Klf4, c-Myc, and Rex1 promotes *Tsix* expression [40], and Rex1 is required for efficient *Tsix* elongation. Factors such as RNF12 (Ring finger protein 12), an X-linked E3 ubiquitin ligase that targets Rex1 for degradation, can activate *Xist* expression even in male mESCs [50] and is essential for XCI initiation [51]. Therefore, *Tsix* and pluripotency factors act synergistically to repress *Xist* in undifferentiated ESCs. 

### 2.4. Loading and Spreading of Xist on the Inactivated X Chromosome

The most extraordinary feature of *Xist* is its ability to “coat” almost an entire X-chromosome. How *Xist* RNAs coat and spread to inactivate the X chromosome remains an open question. Interestingly, naturally occurring or induced X:autosome translocations show different degrees of XCI spread from the X into the autosomal DNA segment, suggesting that specific sequences facilitate spreading [52,53]. These observations prompted investigators to look for potential differences in X-chromosome and autosome sequences. LINE (long interspersed elements) are candidates for mediating this effect due to their higher density on the X chromosome compared to autosomes [54]. The theory proposed by Mary Lyon stated that interspersed repetitive LINE elements act as booster elements to promote spread of *Xist* RNA [54]. A transgenic study in mESCs showed that chromosome regions with higher LINE density are inactivated more efficiently by a *Xist* transgene [53]. A recent study suggests that silenced LINE elements contribute to formation of a heterochromatic compartment initiated by *Xist* RNA and that a particular type of active LINEs may participate in local propagation of the XCI into regions that would otherwise escape [55]. Nonetheless, the exact function of LINE elements in XCI remains to be studied.

In terms of trans-acting factors hnRNPU (also known as SAF-A), a known nuclear scaffold protein, is known to be enriched on the inactive X chromosome [56] and to function in *Xist* RNA loading [57,58]. A recent study showed that YY1, a RNA/DNA binding protein, tethers *Xist* RNA to the inactive X [27]. However, since YY1 binding sites are highly abundant throughout the mammalian genome, it is unclear where the specificity of YY1-specific guidance of *Xist* onto the X-chromosome rather than autosomes lies. Factors mediating this activity remain to be identified. 

### 2.5. Xist Promotes Formation of a Heterochromatic Xi

*Xist* RNA accumulation on Xi leads to chromatin changes, such as DNA hypermethylation, enrichment of macroH2A, loss of the euchromatic mark H3K4-3me and chromosome-wide establishment of heterochromatic mark H3K27-3me and mono ubiquinated H2A (H2AK119u1) [19,59,60,61,62,63]. How can *Xist* lead to these global changes? Since *Xist* coats an entire X, it has long been postulated that *Xist* RNA binds and carries silencing factors, which are deposited as it coats regions during XCI. Thus, much effort has been applied to identifying *Xist*-interacting proteins.

The best-studied *Xist*-interacting trans-factors are Polycomb group proteins. These proteins are highly enriched on the inactive X-chromosome during XCI establishment [60,61,64]. Deletion of some Polycomb proteins, such as Eed, leads to reactivation of the silenced X in extra-embryonic tissues, highlighting their essential role in XCI [65]. However, Polycomb proteins do not seem to affect random X-inactivation in embryos and mESCs, suggesting the existence of a functionally redundant mechanism regulating XCI [66]. Polycomb proteins form two major complexes. One of those, Polycomb Repressive Complex 2 or PRC2, is a histone methyltransferase that trimethylates H3 lysine 27 [67]. Upon recognizing H3K27-3me, another complex, PRC1, is recruited to specific genomic loci to establish ubiquinated histone H2A (H2AK119u1) and compact chromatin for gene silencing. Studies using inducible transgenes showed that PRC2 and PRC1 are localized to Xi in a *Xist* RNA-dependent manner [68,69]. As discussed earlier, it has been suggested that PRC2 is recruited onto Xi by directly interacting with *Xist*/*RepA* lncRNAs via repeat A region [23,26,70]. Furthermore, phosphorylation of Ezh2, the catalytic subunit of PRC2, increases PRC2’s RNA binding ability [71]. Notably, a *Xist* mutant lacking the A-repeats retains the ability to recruit PRC2 and PRC1 to Xi [60,69], indicating that other *Xist* sequences can recruit Polycomb proteins directly or indirectly. Interestingly, in cells with the depletion of a key component of PRC2, some PRC1 proteins can localize to induced *Xist* RNA while others cannot, suggesting *Xist* recruits PRC1 by both PRC2 dependent and independent mechanisms [69]. The PRC2 independent mechanism is supported by a study showing PRC1 can find its genomic targets in PRC2-deficient cells through the interaction with RYBP protein [72]. Surprisingly, trithorax proteins, such as Ash2L, which typically antagonize Polycomb function and are primarily linked to gene activation, also bind *Xist*, suggesting that either Trithorax proteins also function in gene repression or that *Xist* RNA can activate specific genes [58]. 

### 2.6. Perspective

Studies of *Xist* RNA in the past two decades have greatly advanced our understanding of how lncRNAs regulate gene expression epigenetically. However, many questions remain unanswered. For example, it is unclear how *Xist* specifically spreads along the future Xi but not along the activated X. It is also not yet understood how *Xist* RNA interacts with so many different proteins and what determines the specificity of those interactions. And since Polycomb proteins do not function in establishment of XCI in embryos, the mechanisms used to establish the repressive state of the Xi are not yet identified. 

## 3. Imprinted lncRNA *Gtl2*

Genomic imprinting is another dosage regulation mechanism used by mammalian cells. While most autosomal genes are expressed from two parental alleles, some genes are expressed monoallelically in a parent-of-origin manner. This epigenetic phenomenon is termed imprinting. To date, over 100 imprinted genes have been validated in mouse (see the Mouse Imprinting Catalog: http://www.mousebook.org/catalog.php?catalog=imprinting). 

Like X-inactivation, genomic imprinting is tightly controlled by epigenetic mechanisms such as DNA methylation and chromatin modification [73]. Interestingly, within a cluster in which imprinted genes are co-regulated, at least one gene encodes a lncRNA, suggesting the importance of lncRNA in regulating this process [74]. Indeed, some imprinted lncRNAs, such as Air and Kcnq1OT1, have been shown to interact with chromatin modifiers to silence reciprocally imprinted protein coding genes [75,76,77]. Here, we will use the imprinted *Dlk1-Dio3* locus as an example to assess the functional role of lncRNAs in control of imprinting and discuss possible mechanisms used by *Gtl2* (also named *Meg3*), the maternally expressed lncRNA at the *Dlk1-Dio3* locus, to regulate imprinting at this locus. 

### 3.1. Discovery of Gtl2

*Gtl2* was discovered following an insertional mutagenesis gene trap screen to isolate genes differentially regulated during mouse embryonic development [78]. The gene trap insertion at a region 2.3 kb upstream from *Gtl2* promoter caused dwarfism in mouse offspring only through paternal inheritance, not via maternal transmission [78,79]. Subsequent isolation of the *Gtl2* gene suggested that its product was a non-coding RNA (ncRNA) based on the observation that the *Gtl2* contained no significant open reading frames [79]. *Gtl2* was mapped to a region of mouse chromosome 12 (12qF1) thought to contain imprinted genes, due to the observation that in that region uniparental disomy led to embryonic lethality [80]. The paternal-specific growth defect emerging from the insertional mutation plus observation of decreased *Gtl2* expression levels in parthenogenetic embryos led to the hypothesis that *Gtl2* is paternally imprinted [79]. However, soon after, several independent studies demonstrated that *Gtl2* is in fact expressed exclusively from the maternal allele [81,82,83]. The growth phenotype observed following the insertional mutagenesis screen was later suggested to result from perturbations in imprinting of the whole locus, particularly the down regulation of several paternally expressed protein coding genes [84,85].

**Figure 2:**
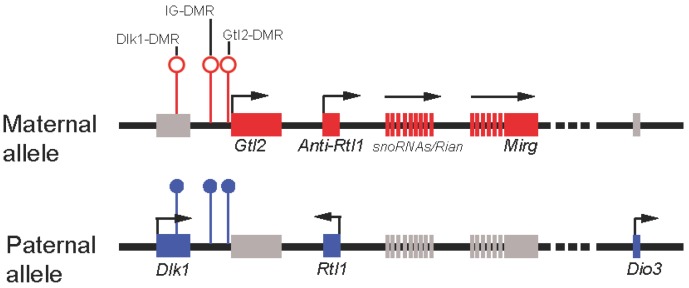
Schematic representation of the mouse imprinted locus *Dlk1-Dio3*. Red rectangles represent maternally-expressed genes, and blue represent paternally-expressed genes. Gray rectangles represent repressed genes. Open circles represent unmethylated DMRs, and filled represent methylated DMRs.

Not long after the discovery of *Gtl2*, other imprinted genes that form a cluster and are co-regulated with *Gtl2* were also identified [81,83,86,87,88,89]. Interestingly, genes preferentially expressed from the paternally-inherited chromosome are all protein-coding genes, including *Dlk1*, *Rtl1*, and *Dio3* (Figure 2), whereas genes expressed maternally all encode ncRNAs, namely, *Gtl2*, *Anti-Rtl1*, *Rian*, *Mirg*, and a large cluster of multiple snoRNAs/microRNAs (Figure 2). All maternally-expressed ncRNAs in this locus are transcribed in the same orientation. This combined with tissue-specific expression of those ncRNAs suggested coordinated expression of the maternally-expressed genes at this locus [90]. It has also been suggested that all of those ncRNAs, with the exception of *Gtl2*, may come from a long polycistronic transcript [90]. Imprinting is typically regulated by a *cis*-element called the differentially methylated region (DMR). Three DMRs, located between *Dlk1* and *Gtl2* (hence intergenic DMR or IG-DMR), at the *Gtl2* promoter, and downstream of *Dlk1* have been identified [91]. While IG-DMR has been shown to regulate the imprinting status of all genes at this locus, the function of the other DMRs remains poorly understood. 

### 3.2. Gtl2 Function

#### 3.2.1. Gtl2 Regulates Genomic Imprinting

Genetic evidence from *Gtl2* knockout mice suggests that *Gtl2* regulates genomic imprinting. One study showed that mice with maternal deletion (300 bp of the promoter and exons 1–5) of the *Gtl2* gene show bidirectional loss of imprinting of all genes in the *Dlk1-Dio3* locus, whereas no effect was detected when the deletion was transmitted paternally, suggesting that *Gtl2* controls genomic imprinting at this locus [92]. *Gtl2* knockout mice phenocopy IG-DMR knockout mice [92,93], implying that *Gtl2* (and/or its downstream ncRNAs) potentially regulates *Dlk1-Dio3* imprinting. Indeed, the same study showed that maternal deletion of *Gtl2* resulted in increased IG-DMR methylation on the maternal allele [92]. Thus, *Gtl2* and/or its downstream ncRNAs may function to maintain the unmethylated status of the maternal IG-DMR through an unknown mechanism. Interestingly, an earlier study deleting ~4Kb of the *Gtl2* promoter plus exons 1–5 yielded significantly different results [94]. The authors did not observe a change in DNA methylation or the imprinted status of any genes within this region when the deletion was transmitted maternally. However, the aberrant imprinted status of *Dlk1*, *Rian*, and *Mirg* was detected when deletion mutation was transmitted paternally. The difference between these two studies could lie in the variable size of the deletion of the *Gtl2* promoter (300 bp *vs*.~4 Kb). Since the region flanking the *Gtl2* promoter is a well-established DMR, these discrepant results suggest a functional role for the *Gtl2* DMR and underscore the importance of studying *Gtl2* function using approaches that minimally perturb the DMR, such as RNAi. 

#### 3.2.2. Gtl2 Regulates Cellular Reprogramming

A recent study showed that mouse induced Pluripotent Stem Cells (iPSCs) in which *Gtl2* and its downstream ncRNAs were aberrantly silenced contributed poorly to chimaeras and failed to generate mice in which every cell is contributed by an iPSC (all-iPSC mice) [95]. In contrast, iPSCs showing normal expression of lncRNAs contributed to high-grade chimaeras and generated viable all-iPSC mice [95]. Further investigation of possible mechanisms underlying repression of maternally imprinted genes revealed that in the iPSCs showing aberrant silencing of lncRNAs from this locus, the maternal IG-DMR was hypermethylated and active histone marks such as H3/H4 acetylation and H3K4 methylation were absent at the *Gtl2* promoter [95]. In a subsequent publication, these authors reported that ascorbic acid facilitates cellular reprogramming by preventing silencing of *Gtl2* and downstream lncRNAs [96]. These findings highlight a role for *Gtl2* in preserving the developmental potential of iPSCs during reprogramming.

#### 3.2.3. Gtl2 Functions in Tumorigenesis

Loss of *GTL2/MEG3* is seen in many tumor types, including human pituitary adenoma, brain tumors, and liver tumors [97,98,99]. *GTL2/MEG3* overexpression inhibits tumor cell proliferation *in vitro* [98,99,100,101,102]. Interestingly, comparison of gene expression profiles in brain between *Gtl2* knockout and wild-type mice revealed an upregulation of genes correlated with angiogenesis in knockout mouse [103], suggesting *Gtl2* may function as a tumor suppressor in part by inhibiting angiogenesis. Furthermore, it was suggested that a postulated tumor-suppressing function of *Gtl2* might stem from its ability to induce p53 accumulation, thereby activating expression of some p53 target genes [99,100,101]. 

### 3.3. Molecular Mechanisms Potentially Underlying Gtl2 Regulation of the Dlk1-Dio3 Locus

lncRNAs employ diverse mechanisms to regulate gene expression at both the transcriptional and post-transcriptional level (reviewed in [104]). Here, we assess possible molecular mechanisms used by *Gtl2* to regulate genes within the *Dlk1-Dio3* locus. The modes of regulation considered are not mutually exclusive: *Gtl2* function might be exerted through combinatorial mechanisms or carried out differentially in response to specific environmental cues.

#### 3.3.1. Gtl2 Maintains Dlk1-Dio3 Imprinting By Protecting the Maternal IG-DMR from Methylation

**Figure 3:**
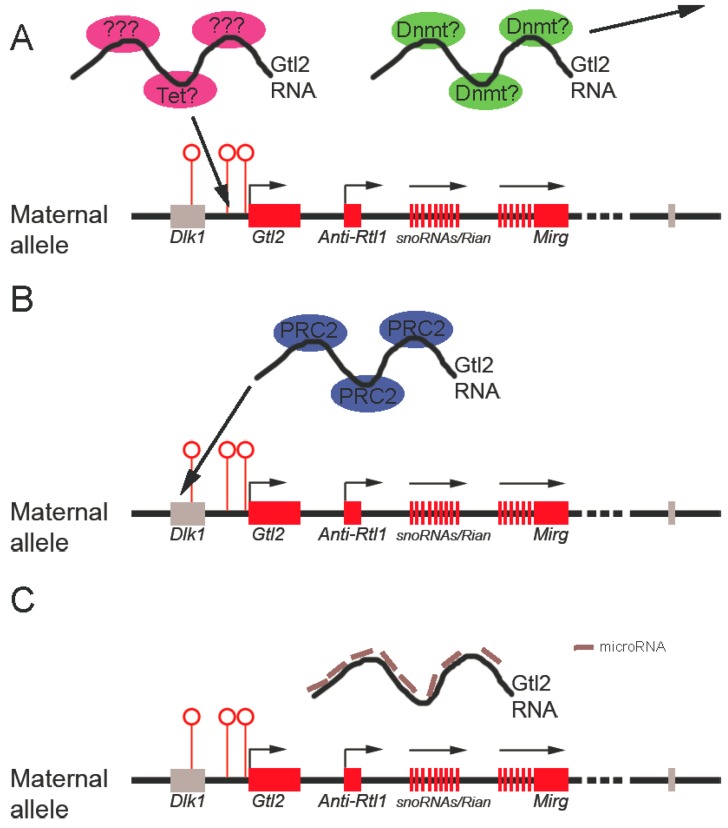
Possible mechanisms underlying *Gtl2* regulation of the imprinted *Dlk1-Dio3* locus. **A.** *Gtl2* may function to maintain the unmethylated status of the IG-DMR by titrating away DNA methyltransferases (Dnmt in the figure) and/or by recruiting DNA demethylation machinery (such as Tet or unknown proteins). **B.** *Gtl2* could silence *Dlk1* expression by recruiting PRC2 complexes to the *Dlk1* genomic region. **C.** Finally, *Gtl2* may serve as competitive endogenous RNA (ceRNA) to keep maternally-expressed genes active by titrating away inhibitory microRNAs targeting those genes. Red rectangles represent maternally-expressed genes. Gray rectangles represent repressed genes. Open circles represent unmethylated DMRs.

Maintaining the proper differential methylation status of the germline DMR is crucial for mono-allelic expression within an imprinting cluster. The observation that targeted deletion of the IG-DMR from the mouse *Dlk1-Dio3* locus caused loss of imprinting when the deletion occurred at the maternal rather than the paternal allele demonstrated that the maternally-inherited unmethylated DMR is essential for the imprinting status of the locus [93]. That *Gtl2* knockdown results in hypermethylation of the maternal IG-DMR suggests that *Gtl2* is required to maintain its unmethylated status. Potential mechanisms include *Gtl2* RNA interaction with and sequestration of positive DNA methylation regulators (such as DNA methyltransferases) or by *Gtl2* actively recruiting negative regulators of DNA demethylation (such as Tet proteins) to keep IG-DMR unmethylated (Figure 3A). These mechanisms are not yet known. 

#### 3.3.2. Gtl2 silences Gene Expression through Recruitment of Chromatin Modifying Machinery

The role of histone modification in regulating imprinting of the *Dlk1-Dio3* locus is less studied than that of DNA methylation. Nevertheless, one study showed that in midgestation mouse embryos, differential histone acetylation occurred between the maternal and paternal alleles of the *Gtl2*-DMR, but not of the IG-DMR [105]. The *Gtl2*-DMR of the active maternal allele was hyperacetylated on H3 and H4, while the silent paternal allele showed a low level of acetylation of both histones. A more recent study that characterized the allele-specific epigenetic profile of the *Gtl2*-DMR in greater detail confirmed the allele-specific acetylation pattern and revealed enrichment of the active histone methylation mark (H3K4 methylation) in the maternal allele in midgestation mouse embryos [106]. It is not clear whether this differential histone modification is instructive for imprinting, or simply reflects the transcriptional state of the genes. 

Our recent studies demonstrate that *Gtl2* directly binds PRC2, and that *Gtl2* knockdown in mouse ESCs results in a reduced Ezh2 occupancy at the *Dlk1* promoter and increased *Dlk1* expression [107]. This data suggests that *Gtl2* can silence gene expression from the maternal allele of the *Dlk1-Dio3* locus through chromatin modification (Figure 3B). It is currently unclear that how *Gtl2* recruits Ezh2 to chromatin and whether *Gtl2* uses the same mechanism to silence genes other than *Dlk1* within the same imprinted locus.

#### 3.3.3. Gtl2 Acts as a ceRNA to Maintain Expression of Maternally-Expressed ncRNAs

The observation that *Gtl2* deletion abolishes expression of downstream lncRNAs and that expression of these RNAs is coordinated suggests that *Gtl2* functions to maintain expression of other maternally-expressed ncRNAs from the *Dlk1-Dio3* locus [90,93]. The recently proposed “competitive endogenous RNA” (ceRNA) theory proposes that RNA transcripts that contain sequences similar to microRNA response elements (MREs) may compete with that pool of microRNAs and alter their availability in cells, thus modulating the level of transcripts targeted by those microRNAs [108]. It has also been suggested that ncRNAs may serve as more efficient ceRNAs than protein-coding RNAs because the binding of microRNA to lncRNAs are not interfered by translation [109]. Indeed, a recent study showed that a muscle-specific lincRNA, linc-MD1, can act as a sponge to titrate away miR-133 and regulate the expression of key transcription factors governing muscle differentiation, suggesting the feasibility of this model [110]. Therefore, *Gtl2* may serve as a ceRNA to titrate away microRNAs targeting other maternally-expressed transcripts at the same locus (Figure 3C). However, as yet, no study has analyzed potential MREs shared between *Gtl2* and those maternally-expressed transcripts. It will be of interest to determine whether lncRNAs regulate each other through this novel mechanism. 

## 4. Conclusions

Since the completion of the Human Genome Project, our perception of the mammalian genome has undergone a dramatic shift. The number of non protein-coding transcripts identified over the past decade has increased exponentially. We now realize that lncRNA is far from the “dark matter” of the genome, but instead plays a critical function in gene regulation. Since XCI and genomic imprinting are two well-defined molecular activities, lncRNAs from XIC or imprinted loci provide ideal systems to understand epigenetic control of how genes are regulated. The mechanisms identified will likely be applicable to newly discovered lncRNAs.
